# A deterministic analysis of an age-sex-structured model for malaria transmission dynamics

**DOI:** 10.1016/j.ijregi.2024.100478

**Published:** 2024-10-23

**Authors:** Marie Aimée Uwineza, Joseph Nzabanita, Innocent Ngaruye, Mouhamadou Bamba Sylla

**Affiliations:** a Department of Mathematics, School of Science, College of Science and Technology, University of Rwanda, Rwanda; b AIMS Research and Innovation Center, African Institute forMathematical Sciences, Rwanda

**Keywords:** Malaria, Basic reproduction number, Stability and sensitivity analysis

## Abstract

*Objectives:* Children younger than 5 years and women, especially pregnant women, are at high risk of malaria and death because of their weak immunity and exposure to mosquitoes. Several studies have considered only the age-structured model and other factors but have not considered sex. The objective of this work is to develop and analyze the malaria transmission model including this structure, to contribute to existing measures and mechanisms to eradicate malaria in Rwanda. *Methods:* A dynamic malaria transmission model considering age and sex structure was developed and analyzed. To study the dynamics of disease, the basic reproduction number was analytically computed and numerically estimated, and the normalized forward sensitivity index was used to highlight its sensitive parameters. *Results:* The most positive sensitive parameters in the model are the force of infection and the infection rates for vectors and female humans aged 5 years or older. The most negative sensitive parameters are the per capita death rates for vectors and humans. *Conclusion:* To control the spread of malaria in Rwanda, the biting and infection rates should be decreased. Therefore, women must comply with government measures against malaria and educate children about it.

## Highlights

• Malaria is a life threatening mosquito-borne disease and it constitutes a global health concern
• Development of Mathematical model for malaria disease taking into account age-gender stratification of the population
• Malaria model analysis
• Malaria transmission control strategies

## 1 Introduction

Malaria is a significant public health concern worldwide. Global health continues to be severely impacted by malaria, one of the deadliest and oldest diseases known to humanity, particularly in tropical and subtropical areas. It is caused by *Plasmodium* parasites transmitted through the bites of infected *Anopheles* mosquitoes. Malaria poses a difficult challenge to healthcare systems, economies, and communities worldwide. Because of the millions of malaria cases and deaths and different preventive measures and strategies taken to eradicate it, in 2015 the World Health Organization aimed to reduce malaria cases and deaths by 40% by 2020 but failed to achieve this, and there were 627,000 malaria deaths and 241 million malaria cases in 2020, which correspond to increases of 7% in malaria deaths and 12% in malaria cases worldwide compared with 2015 [1].

The World Health Organization reported that in 2019 there were 229 million malaria cases and 409,000 malaria deaths worldwide, where two-thirds of these deaths were deaths of children [2]. The report indicated that children younger than 5 years have immunity that is not yet developed to withstand malaria and other diseases, which is why they are affected and killed by malaria to a greater extent than adults.

Mojeeb et al. [3] pointed out that pregnant women have weak immunity and they are more exposed because of their responsibilities of taking care of their families through cooking, washing clothes, and agricultural activities, where women are closer to many growing herbs and stagnant pools of water, which are fruitful breeding grounds for *Anopheles* mosquitoes, which directly transmit malaria [4], [5]. Therefore, for biological and social reasons, women, particularly pregnant women, and children are at the greatest risk of contracting malaria both in high-malaria-endemic and in low-malaria-endemic areas. A study performed in Uganda found that females accounted for almost twice as many cases of malaria as males, and the highest burden of malaria was in women older than 39 years compared with males [6].

From these facts, there is a need to study the age-sex-structured model of malaria transmission to identify and provide advice on malaria preventive and control measures and recommend potential intervention mechanisms and methods to fight against the transmission of malaria in Rwanda, especially among women and children younger than 5 years. Even though the Rwanda *Health Sector Annual Performance Report* for 2020-2021 [7] concluded that there had been a significant decrease in malaria cases and deaths from 2017 to 2021 because of the efforts and strategies taken and implemented by the Government of Rwanda to eradicate malaria, we cannot lower our our guard because malaria still kills many people and also globally malaria continues to ravage societies, especially in Africa.

Several studies on malaria transmission and control have considered the age-structured model and other factors [8], [9], [10], [11] but have not considered sex, and women are a large part of the population who are at high risk of malaria because of their responsibilities that make them more exposed to the high biting rate of mosquitoes than men. The objective of this work is to develop and analyze the malaria transmission model taking into account the age-sex structure of the population and to recommend potential interventions, mechanisms, and methods to fight against the prevalence of malaria in Rwanda.

## 2 Model formulation and analysis

### 2.1 Model formulation

We formulated a model identical to that of Adawe and Lope [9], where the total human population $N_{h}$ is divided into four compartments, which are the susceptible class ($S_{h}$), the exposed class ($E_{h}$), the infectious class ($I_{h}$), and the recovered class ($R_{h}$). The susceptible class is composed of humans who are likely to develop a disease after exposure to the infectious agent. The exposed class comprises humans who are carriers of a disease but who may not show symptoms and present with illness. Susceptible individuals move to the exposed class upon infection, via biting by infectious vectors [12]. When humans develop symptoms and can transmit the disease to susceptible vectors, they move from the exposed class to the infectious class. The recovered class includes the human population that recovered from disease as a result of the effect of treatment that removes parasites from the bloodstream and that acquired immunity.

The main difference between our model and that of Adawe and Lope [9] is that we have included the exposed class in the model from Kalula et al. [10], and we stratified our population not only by age but also by sex (see Tables 1 and 2 for descriptions of the variables and parameters used in the model). Taking into account the age-sex difference in malaria cases and mortality, we incorporated it in all compartments. The total human population is given by $N_{h}=S_{mb}+E_{mb}+I_{mb}+R_{mb}+S_{ma}+E_{ma}+I_{ma}+R_{ma}+S_{fb}+E_{fb}+I_{fb}+R_{fb}+S_{fa}+E_{fa}+I_{fa}+R_{fa}$, with subscripts mb and ma for males younger than 5 years and males aged 5 years or older, respectively, and subscripts fb and fa for females younger than 5 years and females aged 5 years or older, respectively. Hence, the total human population $N_{h}$ is divided into 16 compartments. Likewise, the vector population is divided into three classes: susceptible, exposed, and infectious. There is no recovered class for vectors because of their short life span, and the total vector population is given by $N_{v}=S_{v}+E_{v}+I_{v}$. The purpose of developing the deterministic model is to analyze the effect of several interventions to control and manage the spread of malaria.

**Table 1:** Description of variables used in the model.

Variable	Description
$S_{mb}$	Susceptible male children younger than 5 years
$S_{ma}$	Susceptible males aged 5 years or older
$S_{fb}$	Susceptible female children younger than 5 years
$S_{fa}$	Susceptible females aged 5 years or older
$E_{mb}$	Exposed male children younger than 5 years
$E_{ma}$	Exposed males aged 5 years or older
$E_{fb}$	Exposed female children younger than 5 years
$E_{fa}$	Exposed females aged 5 years or older
$I_{mb}$	Infectious male children younger than 5 years
$I_{ma}$	Infectious males aged 5 years or older
$I_{fb}$	Infectious female children younger than 5 years
$I_{fa}$	Infectious females aged 5 years or older
$R_{mb}$	Recovered male children younger than 5 years
$R_{ma}$	Recovered males aged 5 years or older
$R_{fb}$	Recovered female children younger than 5 years
$R_{fa}$	Recovered females aged 5 years or older
$S_{v}$	Susceptible vectors
$E_{v}$	Exposed vectors
$I_{v}$	Infectious vectors

**Table 2:** Description of parameters used in the model.

Parameter	Description
$\lambda_{h}$	Per capita birth rate for humans
$\lambda_{v}$	Per capita birth rate for vectors
$\mu_{h}$	Per capita death rate for humans
$\mu_{v}$	Per capita death rate for vectors
τ	Rate of maturation of children to adults
$\psi_{i}$(i=mb,ma,fb,fa,v)	Infection rate for susceptible four human strata and vectors
$\alpha_{i}$(i=mb,ma,fb,fa,v)	Force of infection for susceptible four human strata and vectors
$\theta_{i}$(i=mb,ma,fb,fa)	Rate of exposed humans who may recover without developing
	symptoms and without acquiring immunity and may be transferred back
	to the susceptible class
$\phi_{i}$(i=mb,ma,fb,fa,v)	Rate of progression from the exposed class to the infectious class for four human
	strata and vectors
$\xi_{i}$(i=mb,ma,fb,fa)	Rate of infectious humans who may recover without
	acquiring immunity and be transferred back to the susceptible class
$\sigma_{i}$(i=mb,ma,fb,fa)	Malaria-caused death rate for four human strata
$\zeta_{i}$(i=mb,ma,fb,fa)	Rate of recovery of four human strata from malaria after
	acquisition of temporary immunity
$\gamma_{i}$(i=mb,ma,fb,fa)	Rate of recovered people who may lose immunity and move
	back to the susceptible class

We now explain the model dynamics for the first compartments of males younger than 5 years. For the other three strata—males aged 5 years or older, females younger than 5 years, and females aged 5 years or older—the dynamics are similar. Susceptible male children younger than 5 years $S_{mb}$ are recruited through birth at per capita rate $\lambda_{h}$ and diminish through natural death $\mu_{h}$ or they mature and join the corresponding adult compartment at rate τ. Likewise, they reduce in number when they are bitten by an infectious vector $I_{v}$ and become infected, and then move to the exposed class $E_{mb}$ at rate $\psi_{mb}\alpha_{mb}$. Within this phase, they cannot transmit the disease to susceptible vectors as they do not have gametocytes. They leave this compartment through natural death $\mu_{h}$ or becoming mature and joining the corresponding adult compartment $E_{ma}$ or recover naturally with temporal immunity; they may lose this immunity and return to the susceptible class $S_{mb}$ at rate $\theta_{mb}$. A portion of these exposed male children may develop symptoms and move to the infectious class $I_{mb}$ at rate $\phi_{mb}$. At this stage, they can infect susceptible vectors $S_{v}$ since they have gametocytes in their bloodstream. These children leave the infectious class through natural death $\mu_{h}$ or through death due to malaria parasites $\sigma_{mb}$ or they may acquire temporal immunity, which can be lost and, as a result, they move back to the susceptible class $S_{mb}$ at rate $\xi_{mb}$. Others may recover through treatment and move to the recovered class at rate $\zeta_{mb}$. In the recovered class, they leave through natural death μ or through their losing immunity and return to the susceptible class at rate $\gamma_{mb}$.

Susceptible vectors $S_{v}$ are recruited through birth at per capita rate $\lambda_{v}$. They become infected upon biting infectious humans $I_{mb},I_{ma},I_{fb},I_{fa}$ and move to the exposed class $E_{v}$ at rate $\psi_{v}\alpha_{v}$. In the exposed class, they do not have sporozoites in their salivary glands, so they cannot transmit the disease. Once gametocytes develop and change into sporozoites, the vectors become infectious, and therefore they move to the infectious class at rate $\phi_{v}$. At this stage, they can infect susceptible humans, and the dynamics restarts. Because of the short life span of vectors, the recovered class is not considered and there is no induced death in vectors since infection is not harmful to them, and all vectors die naturally at rate $\mu_{v}$.

### 2.2 Model assumptions

(a) The disease-induced death rate is different between males and females and between children and adults (i.e., $\sigma_{mb}>\sigma_{ma}$ and $\sigma_{fb}>\sigma_{fa}$).
(b) In the exposed, infectious, and recovered compartments, humans could acquire immunity naturally, which can be lost and, as a result, they return to the susceptible class.
(c) The total human and vector populations vary over time, and vectors never recover from infection because infection is not harmful to them.

**Fig. 1:**
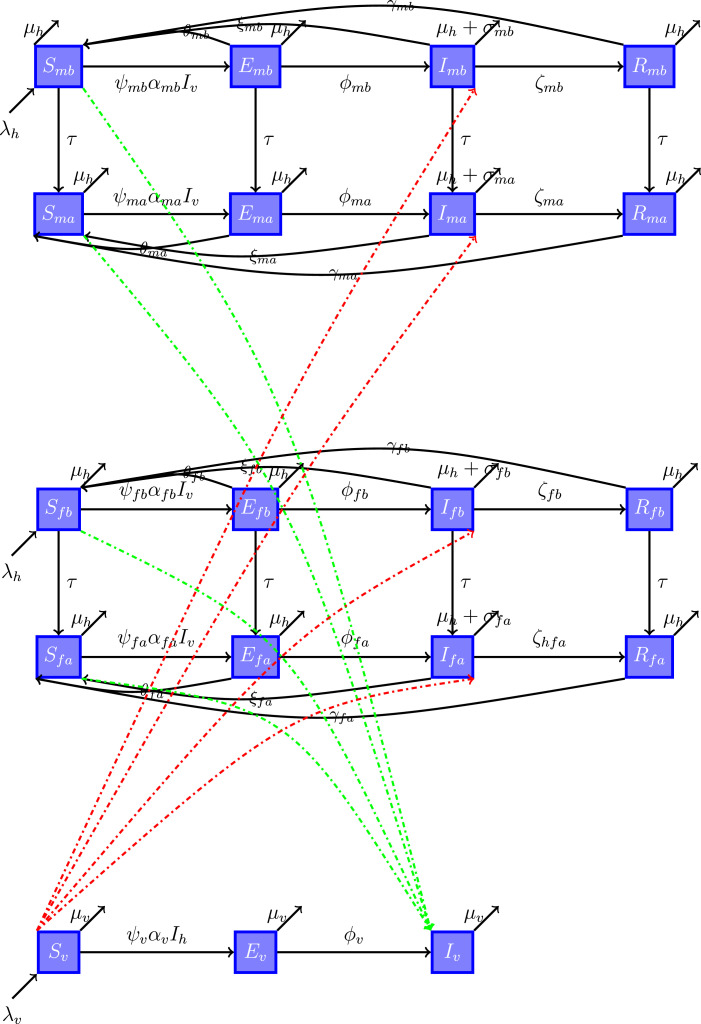
Model flow diagram of malaria dynamics in the four age-sex strata and vectors.

A model flow diagram is shown in Figure 1.

The differential equations are as follows:

(1) $$\begin{array}{cc} \frac{dS_{mb}}{dt}= & \lambda_{h}N_{h}+\theta_{mb}E_{mb}+\xi_{mb}I_{mb}+\gamma_{mb}R_{mb}-\left(\mu_{h}+\tau +\psi_{mb}\alpha_{mb}I_{v}\right)S_{mb}, \end{array}$$

(2) $$\begin{array}{cc} \frac{dS_{ma}}{dt}= & \tau S_{mb}+\theta_{ma}E_{ma}+\xi_{ma}I_{ma}+\gamma_{ma}R_{ma}-\left(\mu_{h}+\psi_{ma}\alpha_{ma}I_{v}\right)S_{ma}, \end{array}$$

(3) $$\begin{array}{cc} \frac{dS_{fb}}{dt}= & \lambda_{h}N_{h}+\theta_{fb}E_{fb}+\xi_{fb}I_{fb}+\gamma_{fb}R_{fb}-\left(\mu_{h}+\tau +\psi_{fb}\alpha_{fb}I_{v}\right)S_{fb}, \end{array}$$

(4) $$\begin{array}{cc} \frac{dS_{fa}}{dt}= & \tau S_{fb}+\theta_{fa}E_{fa}+\xi_{fa}I_{fa}+\gamma_{fa}R_{fa}-\left(\mu_{h}+\psi_{fa}\alpha_{fa}I_{v}\right)S_{fa}, \end{array}$$

(5) $$\begin{array}{cc} \frac{dE_{mb}}{dt}= & \psi_{mb}\alpha_{mb}I_{v}S_{mb}-\left(\mu_{h}+\theta_{mb}+\tau +\phi_{mb}\right)E_{mb}, \end{array}$$

(6) $$\begin{array}{cc} \frac{dE_{ma}}{dt}= & \psi_{ma}\alpha_{ma}I_{v}S_{ma}+\tau E_{mb}-\left(\mu_{h}+\theta_{ma}+\phi_{ma}\right)E_{ma}, \end{array}$$

(7) $$\begin{array}{cc} \frac{dE_{fb}}{dt}= & \psi_{fb}\alpha_{fb}I_{v}S_{fb}-\left(\mu_{h}+\theta_{fb}+\tau +\phi_{fb}\right)E_{fb}, \end{array}$$

(8) $$\begin{array}{cc} \frac{dE_{fa}}{dt}= & \psi_{fa}\alpha_{fa}I_{v}S_{fa}+\tau E_{fb}-\left(\mu_{h}+\theta_{fa}+\phi_{fa}\right)E_{fa}, \end{array}$$

(9) $$\begin{array}{cc} \frac{dI_{mb}}{dt}= & \phi_{mb}E_{mb}-\left(\left(\mu_{h}+\sigma_{mb}\right)+\xi_{mb}+\tau +\zeta_{mb}\right)I_{mb}, \end{array}$$

(10) $$\begin{array}{cc} \frac{dI_{ma}}{dt}= & \phi_{ma}E_{ma}+\tau I_{mb}-\left(\left(\mu_{h}+\sigma_{ma}\right)+\xi_{ma}+\zeta_{ma}\right)I_{ma}, \end{array}$$

(11) $$\begin{array}{cc} \frac{dI_{fb}}{dt}= & \phi_{fb}E_{fb}-\left(\left(\mu_{h}+\sigma_{fb}\right)+\xi_{fb}+\tau +\zeta_{fb}\right)I_{fb}, \end{array}$$

(12) $$\begin{array}{cc} \frac{dI_{fa}}{dt}= & \phi_{fa}E_{fa}+\tau I_{fb}-\left(\left(\mu_{h}+\sigma_{fa}\right)+\xi_{fa}+\zeta_{fa}\right)I_{fa}, \end{array}$$

(13) $$\begin{array}{cc} \frac{dR_{mb}}{dt}= & \zeta_{mb}I_{mb}-\left(\mu_{h}+\gamma_{mb}+\tau \right)R_{mb}, \end{array}$$

(14) $$\begin{array}{cc} \frac{dR_{ma}}{dt}= & \zeta_{ma}I_{ma}+\tau R_{mb}-\left(\mu_{h}+\gamma_{ma}\right)R_{ma}, \end{array}$$

(15) $$\begin{array}{cc} \frac{dR_{fb}}{dt}= & \zeta_{fb}I_{fb}-\left(\mu_{h}+\gamma_{fb}+\tau \right)R_{fb}, \end{array}$$

(16) $$\begin{array}{cc} \frac{dR_{fa}}{dt}= & \zeta_{fa}I_{fa}+\tau R_{fb}-\left(\mu_{h}+\gamma_{fa}\right)R_{fa}, \end{array}$$

(17) $$\begin{array}{cc} \frac{dS_{v}}{dt}= & \lambda_{v}N_{v}-\left(\mu_{v}+\psi_{v}\alpha_{v}I_{h}\right)S_{v}, \end{array}$$

(18) $$\begin{array}{cc} \frac{dE_{v}}{dt}= & \psi_{v}\alpha_{v}I_{h}S_{v}-\left(\mu_{v}+\phi_{v}\right)E_{v}, \end{array}$$

(19) $$\begin{array}{cc} \frac{dI_{v}}{dt}= & \phi_{v}E_{v}-\mu_{v}I_{v}. \end{array}$$

### 2.3 Model analysis

To analyze the model, we follow the work of Mwasunda et al. [13] and Tumwiine et al. [14]

#### 2.3.1 Positivity of the model

From the model equations (1)-(19), we have the following property:

$$\begin{array}{cc} \frac{dS_{ma}}{dt}= & \tau S_{mb}+\theta_{ma}E_{ma}+\xi_{ma}I_{ma}+\gamma_{ma}R_{ma}-\left(\mu_{h}+\psi_{ma}\alpha_{ma}I_{v}\right)S_{ma}. \end{array}$$

It follows that

$$\begin{array}{cc} \frac{dS_{ma}}{dt}⩾ & -\left(\mu_{h}+\psi_{ma}\alpha_{ma}I_{v}\right)S_{ma}. \end{array}$$

So

$$\begin{array}{cc} \frac{dS_{ma}}{S_{ma}}⩾ & -(\mu_{h}+\psi_{ma}\alpha_{ma}I_{v})dt. \end{array}$$

After integrating both sides, we obtain

$$\begin{array}{cc} S_{ma}\left(t\right)⩾ & S_{ma}\left(0\right)\text{exp}\left(-\int_{0}^{t}\left(\mu_{h}+\psi_{ma}\alpha_{ma}I_{v}\right)dt\right), \end{array}$$

which is always positive for all t≥0 as $S_{ma}\geq 0.$ Hence, $S_{ma}\left(t\right)⩾0.$ Similarly,

$$\begin{array}{cc} \frac{dS_{mb}}{dt}⩾ & -\left(\mu_{h}+\psi_{mb}\alpha_{mb}I_{v}\right)S_{mb},\\ \frac{dS_{mb}}{S_{mb}}⩾ & -(\mu_{h}+\psi_{mb}\alpha_{mb}I_{v})dt,\\ S_{mb}\left(t\right)⩾ & S_{mb}\left(0\right)\text{exp}\left(-\int_{0}^{t}\left(\mu_{h}+\psi_{mb}\alpha_{mb}I_{v}\right)dt\right), \end{array}$$

which implies that $S_{mb}\left(t\right)⩾0$ as well. By following the same approach for the remaining model equations, we find that $S_{fa}\left(t\right)⩾0$, $S_{fb}\left(t\right)⩾0$, $E_{ma}\left(t\right)⩾0$, $E_{mb}\left(t\right)⩾0$, $E_{fa}\left(t\right)⩾0$, $E_{fb}\left(t\right)⩾0$, $I_{ma}\left(t\right)⩾0$, $I_{mb}\left(t\right)⩾0$, $I_{fa}\left(t\right)⩾0$, $I_{fb}\left(t\right)⩾0$, $R_{ma}\left(t\right)⩾0$, $R_{mb}\left(t\right)⩾0$, $R_{fa}\left(t\right)⩾0$, $R_{fb}\left(t\right)⩾0$, $S_{v}\left(t\right)⩾0$, $E_{v}\left(t\right)⩾0$, and $I_{v}\left(t\right)⩾0.$ Therefore, the solution of the formulated malaria model has non-negative solutions for all of its equations and for all the time horizon.

#### 2.3.2 Invariant region and boundedness

We have split the population into four categories on the basis of an age-sex structure and the vectors, and the following total populations are observed in each category:

$$\begin{array}{cc} N_{ma}= & S_{ma}+E_{ma}+I_{ma}+R_{ma},\\ N_{mb}= & S_{mb}+E_{mb}+I_{mb}+R_{mb},\\ N_{fa}= & S_{fa}+E_{fa}+I_{fa}+R_{fa},\\ N_{fb}= & S_{fb}+E_{fb}+I_{fb}+R_{fb},\\ N_{v}= & S_{v}+E_{v}+I_{v}. \end{array}$$

So

(20) $$\begin{array}{ccc} \frac{dN_{ma}}{dt} & = & \frac{dS_{ma}}{dt}+\frac{dE_{ma}}{dt}+\frac{dI_{ma}}{dt}+\frac{dR_{ma}}{dt}\\ & = & \tau N_{mb}-\mu_{h}N_{ma}-\sigma_{ma}I_{ma},\\ \frac{dN_{mb}}{dt} & = & \frac{dS_{mb}}{dt}+\frac{dE_{mb}}{dt}+\frac{dI_{mb}}{dt}+\frac{dR_{mb}}{dt}\\ & = & \lambda_{h}N_{h}-\left(\mu_{h}+\tau \right)N_{mb}-\sigma_{mb}I_{mb},\\ \frac{dN_{fa}}{dt} & = & \frac{dS_{fa}}{dt}+\frac{dE_{fa}}{dt}+\frac{dI_{fa}}{dt}+\frac{dR_{ma}}{dt}\\ & = & \tau N_{fb}-\mu_{h}N_{fa}-\sigma_{fa}I_{fa},\\ \frac{dN_{fb}}{dt} & = & \frac{dS_{fb}}{dt}+\frac{dE_{fb}}{dt}+\frac{dI_{fb}}{dt}+\frac{dR_{fb}}{dt}\\ & = & \lambda_{h}N_{h}-\left(\mu_{h}+\tau \right)N_{fb}-\sigma_{fb}I_{fb},\\ \frac{dN_{v}}{dt} & = & \frac{dS_{v}}{dt}+\frac{dE_{v}}{dt}+\frac{dI_{v}}{dt}\\ & = & \left(\lambda_{v}-\mu_{v}\right)N_{v}. \end{array}$$

From equations (20), we can show that

(21) $$\begin{array}{ccc} \frac{dN_{ma}}{dt}+\mu_{h}N_{ma} & ⩽ & \tau N_{mb},\\ \frac{dN_{mb}}{dt}+\left(\mu_{h}+\tau \right)N_{mb} & ⩽ & \lambda_{h}N_{h},\\ \frac{dN_{fa}}{dt}+\mu_{h}N_{fa} & ⩽ & \tau N_{fb},\\ \frac{dN_{fb}}{dt}+\left(\mu_{h}+\tau \right)N_{fb} & ⩽ & \lambda_{h}N_{h},\\ \frac{dN_{v}}{dt} & ⩽ & \left(\lambda_{v}-\mu_{v}\right)N_{v}. \end{array}$$

On solving equations (21) by using the integrating factor and by considering the initial solutions for the populations at t=0 to be $N_{ma}\left(0\right)$, $N_{mb}\left(0\right)$, $N_{fa}\left(0\right)$, $N_{fb}\left(0\right)$, and $N_{v}\left(0\right),$ we obtain

(22) $$\begin{array}{ccc} N_{ma}\left(t\right) & ⩽ & \frac{\tau N_{mb}}{\mu_{h}}+\left(N_{ma}\left(0\right)-\frac{\tau N_{mb}}{\mu_{h}}\right)\text{exp}\left(-\mu_{h}t\right),\\ N_{mb}\left(t\right) & ⩽ & \frac{\lambda_{h}N_{h}}{\mu_{h}+\tau }+\left(N_{mb}\left(0\right)-\frac{\lambda_{h}N_{h}}{\mu_{h}+\tau }\right)\text{exp}\left(-(\mu_{h}+\tau )t\right),\\ N_{fa}\left(t\right) & ⩽ & \frac{\tau N_{fb}}{\mu_{h}}+\left(N_{fa}\left(0\right)-\frac{\tau N_{fb}}{\mu_{h}}\right)\text{exp}\left(-\mu_{h}t\right),\\ N_{fb}\left(t\right) & ⩽ & \frac{\lambda_{h}N_{h}}{\mu_{h}+\tau }+\left(N_{fb}\left(0\right)-\frac{\lambda_{h}N_{h}}{\mu_{h}+\tau }\right)\text{exp}\left(-(\mu_{h}+\tau )t\right),\\ N_{v}\left(t\right) & ⩽ & N_{v}\left(0\right)\text{exp}\left(\lambda_{v}-\mu_{v}\right)t. \end{array}$$

The analysis of equations (22) leads to

$$N_{ma}\left(0\right)>\frac{\tau N_{mb}}{\mu_{h}}$$

or

$$N_{ma}\left(0\right)<\frac{\tau N_{mb}}{\mu_{h}},$$

which implies that

$$N_{ma}\left(t\right)⩽T\left(t\right)=\text{max}\left(N_{ma}\left(0\right),\frac{\tau N_{mb}}{\mu_{h}}\right).$$

In the same way,

$$\begin{array}{ccc} N_{mb}\left(0\right) & > & \frac{\lambda_{h}N_{h}}{\left(\mu_{h}+\tau \right)}\;\text{or}\;N_{mb}\left(0\right)<\frac{\lambda_{h}N_{h}}{\left(\mu_{h}+\tau \right)}⟹N_{mb}\left(t\right)⩽P\left(t\right)\\ & = & \text{max}\left(N_{mb}\left(0\right),\frac{\lambda_{h}N_{h}}{\left(\mu_{h}+\tau \right)}\right),\\ N_{fa}\left(0\right) & > & \frac{\tau N_{fb}}{\mu_{h}}\;\text{or}\;N_{ma}\left(0\right)<\frac{\tau N_{fb}}{\mu_{h}}⟹N_{fa}\left(t\right)⩽Q\left(t\right)\\ & = & \text{max}\left(N_{fa}\left(0\right),\frac{\tau N_{fb}}{\mu_{h}}\right),\\ N_{fb}\left(0\right) & > & \frac{\lambda_{h}N_{h}}{\left(\mu_{h}+\tau \right)}\;\text{or}\;N_{fb}\left(0\right)<\frac{\lambda_{h}N_{h}}{\left(\mu_{h}+\tau \right)}⟹N_{fb}\left(t\right)⩽U\left(t\right)\\ & = & \text{max}\left(N_{fb}\left(0\right),\frac{\lambda_{h}N_{h}}{\left(\mu_{h}+\tau \right)}\right),\\ N_{v}\left(t\right) & ⩽ & N_{v}\left(0\right)\text{exp}\left(\lambda_{v}-\mu_{v}\right)t⩽M\left(t\right)<+\infty . \end{array}$$

As $N_{ma}\left(t\right)⩽T\left(t\right)$ and $N_{ma}\left(t\right)=S_{ma}+E_{ma}+I_{ma}+R_{ma},$ we have $S_{ma}\left(t\right)⩽T\left(t\right)$, $E_{ma}\left(t\right)⩽T\left(t\right)$, $I_{ma}\left(t\right)⩽T\left(t\right)$, and $R_{ma}\left(t\right)⩽T\left(t\right).$ We also have $N_{mb}\left(t\right)⩽P\left(t\right)⟹S_{mb}\left(t\right)⩽P\left(t\right)$, $E_{mb}\left(t\right)⩽P\left(t\right)$, $I_{mb}\left(t\right)⩽P\left(t\right)$, and $R_{mb}\left(t\right)⩽P\left(t\right).$ The same procedure results in $N_{fa}\left(t\right)⩽Q\left(t\right)⟹S_{fa}\left(t\right)⩽Q\left(t\right)$, $E_{fa}\left(t\right)⩽Q\left(t\right)$, $I_{fa}\left(t\right)⩽Q\left(t\right)$, $R_{fa}\left(t\right)⩽Q\left(t\right)$, $N_{fb}\left(t\right)⩽U\left(t\right)⟹S_{fb}\left(t\right)⩽U\left(t\right)$, $E_{fb}\left(t\right)⩽U\left(t\right)$, $I_{fb}\left(t\right)⩽U\left(t\right)$ $R_{fb}\left(t\right)⩽U\left(t\right)$, $N_{v}\left(t\right)⩽M\left(t\right)⟹S_{v}\left(t\right)⩽M\left(t\right)$, $E_{v}\left(t\right)⩽M\left(t\right)$, and $I_{v}\left(t\right)⩽M\left(t\right).$

As a result, the solutions of the model equations in the system (1)–(19) are positive and bounded in the invariant region:

(23) $$\begin{array}{ccc} \Omega & = & \{(S_{ma}\left(t\right),E_{ma}\left(t\right),I_{ma}\left(t\right),R_{ma}\left(t\right),S_{mb}\left(t\right),E_{mb}\left(t\right),I_{mb}\left(t\right),R_{mb}\left(t\right),\\ & & S_{fa}\left(t\right),E_{fa}\left(t\right),I_{fa}\left(t\right),R_{fa}\left(t\right),S_{fb}\left(t\right),E_{fb}\left(t\right),I_{fb}\left(t\right),R_{fb}\left(t\right),\\ & & S_{v}\left(t\right),E_{v}\left(t\right),I_{v}\left(t\right))\in R_{+}^{19}:0⩽N_{ma}\left(t\right)⩽T\left(t\right);\\ & & \;0⩽N_{mb}\left(t\right)⩽P\left(t\right);\;0⩽N_{fa}\left(t\right)⩽Q\left(t\right);\;0⩽N_{fb}\left(t\right)⩽U\left(t\right);\;\\ & & 0⩽N_{v}\left(t\right)⩽M\left(t\right)\}. \end{array}$$

Therefore, all initial solutions that start in the neighborhood of Ω will be bounded and converge to their solutions in the invariant region Ω. This shows that the model biologically and epidemiologically makes sense for further analysis and applications.

### 2.4 Basic reproduction number

The basic reproduction number $R_{0}$ is the expected number of secondary cases per primary case in a “virgin” population [15]. It has three threshold values as follows:

(a) $R_{0}>1$: This is an epidemic situation; the disease spreads at a very high rate.
(b) $R_{0}=1$: This is an endemic situation; the disease spreads at a low rate.
(c) $R_{0}<1$: The disease will die out.

Hence, the basic reproduction number can be defined as the number $R_{0}$ such that if it is less than 1, the disease-free equilibrium is locally asymptotically stable, and the disease cannot invade the population. However, if the basic reproduction number is greater than 1, then the disease-free equilibrium is unstable, and invasion is always possible.

The basic reproduction number was analytically solved with the use of the next-generation matrix method described by van den Driessche and Watmough [16]. We define F as a matrix for new infection terms and V as a matrix for transfer terms. F and V are computed at the disease-free equilibrium point.

In addition, F is a non-negative matrix and V is an invertible matrix. Therefore, we compute the inverse $V^{-1}$ of V and the product $FV^{-1}$. Finally, the basic reproduction number $R_{0}$ is given by the spectral radius or maximum eigenvalue of the matrix $FV^{-1}$, i.e.,

$$R_{0}=l\left(FV^{-1}\right).$$

This solution of the basic reproduction number was evaluated at the disease-free equilibrium point. We define a disease-free equilibrium point as a stable state solution where the disease infections do not exist in the population. In this absence of the disease, the infectious compartments become zeros, which implies that their corresponding recovery compartments are zeros too, and therefore the above system has a disease-free equilibrium point written as $X_{0}=\left(S_{mb}^{*},S_{ma}^{*},S_{fb}^{*},S_{fa}^{*},E_{mb}^{,}E_{ma}^{*},E_{fb}^{*},E_{fa}^{*},I_{mb}^{*},I_{ma}^{*},I_{fb}^{*},I_{fa}^{*},R_{mb}^{*},R_{ma}^{*},R_{fb}^{*},R_{fa}^{*},S_{v}^{*},E_{v}^{*},R_{v}^{*}\right)$, where $\left(E_{mb}^{*},E_{ma}^{*},E_{fb}^{*},E_{fa}^{*},I_{mb}^{*},I_{ma}^{*},I_{fb}^{*},I_{fa}^{*},R_{mb}^{*},R_{ma}^{*},R_{fb}^{*},R_{fa}^{*},E_{v}^{*},I_{v}^{*}\right)$ are zeros. After solving the model developed, we obtain the disease-free equilibrium point given by

$$\begin{array}{cc} X_{0}=(S_{ma}^{*}= & \frac{\lambda (\lambda_{h}N_{h})}{\mu_{h}\left(\mu_{h}+\tau \right)},0,0,0,\\ S_{mb}^{*}= & \frac{\lambda_{h}N_{h}}{\mu_{h}+\tau },0,0,0,\\ S_{fa}^{*}= & \frac{\tau \lambda_{h}N_{h}}{\mu_{h}\left(\mu_{h}+\tau \right)},0,0,0,\\ S_{fb}^{*}= & \frac{\lambda_{h}N_{h}}{\mu_{h}+\tau },0,0,0,\\ S_{v}^{*}= & \frac{\lambda_{v}N_{v}}{\mu_{v}+\psi_{v}\alpha_{v}I_{h}},0,0). \end{array}$$

If we arrange equations (1)-(19) according to the compartments that transmit the disease, we obtain the following system of nonlinear equations:

(24) $$\begin{array}{cc} \frac{dE_{mb}}{dt}= & \psi_{mb}\alpha_{mb}I_{v}S_{mb}-\left(\mu_{h}+\theta_{mb}+\tau +\phi_{mb}\right)E_{mb}, \end{array}$$

(25) $$\begin{array}{cc} \frac{dE_{ma}}{dt}= & \psi_{ma}\alpha_{ma}I_{v}S_{ma}+\tau E_{mb}-\left(\mu_{h}+\theta_{ma}+\phi_{ma}\right)E_{ma}, \end{array}$$

(26) $$\begin{array}{cc} \frac{dE_{fb}}{dt}= & \psi_{fb}\alpha_{fb}I_{v}S_{fb}-\left(\mu_{h}+\theta_{fb}+\tau +\phi_{fb}\right)E_{fb}, \end{array}$$

(27) $$\begin{array}{cc} \frac{dE_{fa}}{dt}= & \psi_{fa}\alpha_{fa}I_{v}S_{fa}+\tau E_{fb}-\left(\mu_{h}+\theta_{fa}+\phi_{fa}\right)E_{fa}, \end{array}$$

(28) $$\begin{array}{cc} \frac{dI_{mb}}{dt}= & \phi_{mb}E_{mb}-\left(\left(\mu_{h}+\sigma_{mb}\right)+\xi_{mb}+\tau +\zeta_{mb}\right)I_{mb}, \end{array}$$

(29) $$\begin{array}{cc} \frac{dI_{ma}}{dt}= & \phi_{ma}E_{ma}+\tau I_{mb}-\left(\left(\mu_{h}+\sigma_{ma}\right)+\xi_{ma}+\zeta_{ma}\right)I_{ma}, \end{array}$$

(30) $$\begin{array}{cc} \frac{dI_{fb}}{dt}= & \phi_{fb}E_{fb}-\left(\left(\mu_{h}+\sigma_{fb}\right)+\xi_{fb}+\tau +\zeta_{fb}\right)I_{fb}, \end{array}$$

(31) $$\begin{array}{cc} \frac{dI_{fa}}{dt}= & \phi_{fa}E_{fa}+\tau I_{fb}-\left(\left(\mu_{h}+\sigma_{fa}\right)+\xi_{fa}+\zeta_{fa}\right)I_{fa}, \end{array}$$

(32) $$\begin{array}{cc} \frac{dE_{v}}{dt}= & \psi_{v}\alpha_{v}I_{h}S_{v}-\left(\mu_{v}+\phi_{v}\right)E_{v}, \end{array}$$

(33) $$\begin{array}{cc} \frac{dI_{v}}{dt}= & \phi_{v}E_{v}-\mu_{v}I_{v}, \end{array}$$

(34) $$\begin{array}{cc} \frac{dS_{mb}}{dt}= & \lambda_{h}N_{h}+\theta_{mb}E_{mb}+\xi_{mb}I_{mb}+\gamma_{mb}R_{mb}-\left(\mu_{h}+\tau +\psi_{mb}\alpha_{mb}I_{v}\right)S_{mb}, \end{array}$$

(35) $$\begin{array}{cc} \frac{dS_{ma}}{dt}= & \tau S_{mb}+\theta_{ma}E_{ma}+\xi_{ma}I_{ma}+\gamma_{ma}R_{ma}-\left(\mu_{h}+\psi_{ma}\alpha_{ma}I_{v}\right)S_{ma}, \end{array}$$

(36) $$\begin{array}{cc} \frac{dS_{fb}}{dt}= & \lambda_{h}N_{h}+\theta_{fb}E_{fb}+\xi_{fb}I_{fb}+\gamma_{fb}R_{fb}-\left(\mu_{h}+\tau +\psi_{fb}\alpha_{fb}I_{v}\right)S_{fb}, \end{array}$$

(37) $$\begin{array}{cc} \frac{dS_{fa}}{dt}= & \tau S_{fb}+\theta_{fa}E_{fa}+\xi_{fa}I_{fa}+\gamma_{fa}R_{fa}-\left(\mu_{h}+\psi_{fa}\alpha_{fa}I_{v}\right)S_{fa}, \end{array}$$

(38) $$\begin{array}{cc} \frac{dR_{mb}}{dt}= & \zeta_{mb}I_{mb}-\left(\mu_{h}+\gamma_{mb}+\tau \right)R_{mb}, \end{array}$$

(39) $$\begin{array}{cc} \frac{dR_{ma}}{dt}= & \zeta_{ma}I_{ma}+\tau R_{mb}-\left(\mu_{h}+\gamma_{ma}\right)R_{ma}, \end{array}$$

(40) $$\begin{array}{cc} \frac{dR_{fb}}{dt}= & \zeta_{fb}I_{fb}-\left(\mu_{h}+\gamma_{fb}+\tau \right)R_{fb}, \end{array}$$

(41) $$\begin{array}{cc} \frac{dR_{fa}}{dt}= & \zeta_{fa}I_{fa}+\tau R_{fb}-\left(\mu_{h}+\gamma_{fa}\right)R_{fa}, \end{array}$$

(42) $$\begin{array}{cc} \frac{dS_{v}}{dt}= & \lambda_{v}N_{v}-\left(\mu_{v}+\psi_{v}\alpha_{v}I_{h}\right)S_{v}. \end{array}$$

By using the disease-free equilibrium point obtained, we can now calculate the basic reproduction number using the next-generation matrix:

$$\begin{array}{ccc} F & = & \left[\begin{array}{c} \psi_{mb}\alpha_{mb}S_{mb}I_{v}\\ \psi_{ma}\alpha_{ma}S_{ma}I_{v}\\ \psi_{fb}\alpha_{fb}S_{fb}I_{v}\\ \psi_{fa}\alpha_{fa}S_{fa}I_{v}\\ 0\\ 0\\ 0\\ 0\\ \psi_{v}\alpha_{v}S_{v}I_{h}\\ 0\\ 0\\ 0\\ 0\\ 0\\ 0\\ 0\\ 0\\ 0\\ 0 \end{array}\right]\;\mathrm{and}\\ V & = & \left[\begin{array}{c} \left(\mu_{h}+\theta_{mb}+\tau +\phi_{mb}\right)E_{mb}\\ -\tau E_{mb}+\left(\mu_{h}+\theta_{ma}+\phi_{ma}\right)E_{ma}\\ \left(\mu_{h}+\theta_{fb}+\tau +\phi_{fb}\right)E_{fb}\\ -\tau E_{fb}+\left(\mu_{h}+\theta_{fa}+\phi_{fa}\right)E_{fa}\\ -\phi_{mb}E_{mb}+\left(\left(\mu_{h}+\sigma_{mb}\right)+\xi_{mb}+\tau +\zeta_{mb}\right)I_{mb}\\ -\phi_{ma}E_{ma}-\tau I_{mb}+\left(\left(\mu_{h}+\sigma_{ma}\right)+\xi_{ma}+\zeta_{ma}\right)I_{ma}\\ -\phi_{fb}E_{fb}+\left(\left(\mu_{h}+\sigma_{fb}\right)+\xi_{fb}+\tau +\zeta_{fb}\right)I_{fb}\\ -\phi_{fa}E_{fa}-\tau I_{fb}+\left(\left(\mu_{h}+\sigma_{fa}\right)+\xi_{fa}+\zeta_{fa}\right)I_{fa}\\ \left(\mu_{v}+\phi_{v}\right)E_{v}\\ -\phi_{v}E_{v}+\mu_{v}I_{v}\\ -\lambda_{h}N_{h}-\theta_{mb}E_{mb}-\xi_{mb}I_{mb}-\gamma_{mb}R_{mb}+\left(\mu_{h}+\tau \right)S_{mb}\\ -\tau S_{mb}-\theta_{ma}E_{ma}-\xi_{ma}I_{ma}-\gamma_{ma}R_{ma}+\mu_{h}S_{ma}\\ -\lambda_{h}N_{h}-\theta_{fb}E_{fb}-\xi_{fb}I_{fb}-\gamma_{fb}R_{fb}+\left(\mu_{h}+\tau \right)S_{fb}\\ -\tau S_{fb}-\theta_{fa}E_{fa}-\xi_{fa}I_{fa}-\gamma_{fa}R_{fa}+\mu_{h}S_{fa}\\ -\zeta_{mb}I_{mb}+\left(\mu_{h}+\gamma_{mb}+\tau \right)R_{mb}\\ -\zeta_{ma}I_{ma}-\tau R_{mb}+\left(\mu_{h}+\gamma_{ma}\right)R_{ma}\\ -\zeta_{fb}I_{fb}+\left(\mu_{h}+\gamma_{fb}+\tau \right)R_{fb}\\ -\zeta_{fa}I_{fa}-\tau R_{fb}+\left(\mu_{h}+\gamma_{fa}\right)R_{fa}\\ -\lambda_{v}N_{v}+\mu_{v}S_{v} \end{array}\right]. \end{array}$$

By finding the partial derivative of F and V with respect to the variables $E_{mb}$, $E_{ma}$, $E_{fb}$, $E_{fa}$, $I_{mb}$, $I_{ma}$, $I_{fb}$, $I_{fa}$, $E_{v}$, and $I_{v}$, we obtain matrices F and V:

$$\begin{array}{cc} F= & \left[\begin{array}{cccccccccc} 0 & 0 & 0 & 0 & 0 & 0 & 0 & 0 & 0 & \psi_{mb}\alpha_{mb}S_{mb}^{*}\\ 0 & 0 & 0 & 0 & 0 & 0 & 0 & 0 & 0 & \psi_{ma}\alpha_{ma}S_{ma}^{*}\\ 0 & 0 & 0 & 0 & 0 & 0 & 0 & 0 & 0 & \psi_{fb}\alpha_{fb}S_{fb}^{*}\\ 0 & 0 & 0 & 0 & 0 & 0 & 0 & 0 & 0 & \psi_{fa}\alpha_{fa}S_{fa}^{*}\\ 0 & 0 & 0 & 0 & 0 & 0 & 0 & 0 & 0 & 0\\ 0 & 0 & 0 & 0 & 0 & 0 & 0 & 0 & 0 & 0\\ 0 & 0 & 0 & 0 & 0 & 0 & 0 & 0 & 0 & 0\\ 0 & 0 & 0 & 0 & 0 & 0 & 0 & 0 & 0 & 0\\ 0 & 0 & 0 & 0 & \psi_{v}\alpha_{v}S_{v}^{*} & \psi_{v}\alpha_{v}S_{v}^{*} & \psi_{v}\alpha_{v}S_{v}^{*} & \psi_{v}\alpha_{v}S_{v}^{*} & 0 & 0\\ 0 & 0 & 0 & 0 & 0 & 0 & 0 & 0 & 0 & 0 \end{array}\right], \end{array}$$

$$\begin{array}{cc} V= & [\begin{array}{ccccc} \mu_{h}+\theta_{mb}+\tau +\phi_{mb} & 0 & 0 & 0 & 0\\ -\tau & \mu_{h}+\theta_{ma}+\phi_{ma} & 0 & 0 & 0\\ 0 & 0 & \mu_{h}+\theta_{fb}+\tau +\phi_{fb} & 0 & 0\\ 0 & 0 & -\tau & \mu_{h}+\theta_{fa}+\phi_{fa} & 0\\ -\phi_{mb} & 0 & 0 & 0 & \mu_{h}+\sigma_{mb}+\xi_{mb}+\tau +\zeta_{mb}\\ 0 & -\phi_{ma} & 0 & 0 & -\tau \\ 0 & 0 & -\phi_{fb} & 0 & 0\\ 0 & 0 & 0 & -\phi_{fa} & 0\\ 0 & 0 & 0 & 0 & 0\\ 0 & 0 & 0 & 0 & 0 \end{array}\\ & \begin{array}{ccccc} 0 & 0 & 0 & 0 & 0\\ 0 & 0 & 0 & 0 & 0\\ 0 & 0 & 0 & 0 & 0\\ 0 & 0 & 0 & 0 & 0\\ 0 & 0 & 0 & 0 & 0\\ \mu_{h}+\sigma_{ma}+\xi_{ma}+\zeta_{ma}) & 0 & 0 & 0 & 0\\ 0 & \mu_{h}+\sigma_{fb}+\xi_{fb}+\tau +\zeta_{fb} & 0 & 0 & 0\\ 0 & -\tau & \mu_{h}+\sigma_{fa}+\xi_{fa}+\zeta_{fa} & 0 & 0\\ 0 & 0 & 0 & \mu_{v}+\phi_{v} & 0\\ 0 & 0 & 0 & -\phi_{v} & \mu_{v} \end{array}]. \end{array}$$

To get the basic reproduction number, we computed the spectral radius or maximum eigenvalue of the generation matrix $FV^{-1}$, resulting in the following analytical solution of the basic reproduction number:

(43) $$R_{0}=\sqrt{\frac{\phi_{v}.v\phi_{f_{b}}f_{b}gpq\left(k\tau^{2}+th+s\tau +kt\tau \right)}{\left(gk\mu_{v}+pqstux\right)\left(h+\tau \right)}+\frac{\phi_{v}v\phi_{fa}f_{a}ghpqsu\left(\tau +h\right)}{\left(gk\mu_{v}+pqstux\right)\left(h+\tau \right)}+\frac{\phi_{v}v\phi_{ma}kps\left(ahtu+a\tau +bt\tau u\right)}{\left(gk\mu_{v}+pqstux\right)\left(h+\tau \right)}+\frac{\phi_{v}v\phi_{mb}bgkqstu\left(1+\tau \right)}{\left(gk\mu_{v}+pqstux\right)\left(h+\tau \right)}}.$$

So

(44) $$R_{0}=\sqrt{R_{0v}fb+R_{0v}fa+R_{0v}ma+R_{0v}mb},$$

where

$$\begin{array}{cc} R_{0v}fb= & \frac{\phi_{v}v\phi_{f_{b}}f_{b}gpq\left(k\tau^{2}+th+s\tau +kt\tau \right)}{\left(gk\mu_{v}+pqstux\right)\left(h+\tau \right)},\\ R_{0v}fa= & \frac{\phi_{v}v\phi_{fa}f_{a}ghpqsu\left(\tau +h\right)}{\left(gk\mu_{v}+pqstux\right)\left(h+\tau \right)},\\ R_{0v}ma= & \frac{\phi_{v}v\phi_{ma}kps\left(ahtu+a\tau +bt\tau u\right)}{\left(gk\mu_{v}+pqstux\right)\left(h+\tau \right)},\\ R_{0v}mb= & \frac{\phi_{v}v\phi_{mb}bgkqstu\left(1+\tau \right)}{\left(gk\mu_{v}+pqstux\right)\left(h+\tau \right)}, \end{array}$$

where $b=\phi_{mb}\alpha_{mb}S_{mb}^{*}$, $a=\phi_{ma}\alpha_{ma}S_{ma}^{*}$, $fb=fb\alpha_{fb}S_{fb}^{*}$, $fa=\phi_{fa}\alpha_{fa}S_{fa}^{*}$, $v=\phi_{v}\alpha_{v}\times S_{v}^{*}$, $h=\mu_{h}+\theta_{mb}+\Phi_{mb}$, $g=\mu_{h}+\theta_{ma}+\Phi_{ma}$, $u=\mu_{h}+\theta_{fb}+\tau +\Phi_{fb}$, $k=\mu_{h}+\theta_{fa}+\Phi_{fa}$, $p=\mu_{h}+\sigma_{mb}+\xi_{mb}+\tau +\psi_{mb}$, $q=\mu_{h}+\sigma_{ma}+\xi_{ma}+\psi_{ma}$, $s=\mu_{h}+\sigma_{fb}+\xi_{fb}+\tau +\psi_{fb}$, $t=\mu_{h}+\sigma_{fa}+\xi_{fa}+\psi_{fa}$, and $x=\mu_{v}+\Phi_{v}$.

## 3 Stability analysis of equilibrium points

### 3.1 Global stability of the disease-free equilibrium point

The disease-free equilibrium point was found to be given by

$$\begin{array}{cc} X_{0}=(S_{ma}^{*}= & \frac{\lambda (\lambda_{h}N_{h})}{\mu_{h}\left(\mu_{h}+\tau \right)},0,0,0,\\ S_{mb}^{*}= & \frac{\lambda_{h}N_{h}}{\mu_{h}+\tau },0,0,0,\\ S_{fa}^{*}= & \frac{\tau \lambda_{h}N_{h}}{\mu_{h}\left(\mu_{h}+\tau \right)},0,0,0,\\ S_{fb}^{*}= & \frac{\lambda_{h}N_{h}}{\mu_{h}+\tau },0,0,0,\\ S_{v}^{*}= & \frac{\lambda_{v}N_{v}}{\mu_{v}+\psi_{v}\alpha_{v}I_{h}},0,0). \end{array}$$

**Theorem:** The disease-free equilibrium point is stable when $R_{0}<1$ and unstable when $R_{0}>0.$ For the formulated model, the stability analysis of the disease-free equilibrium point is done by our taking into consideration the work of Castillo-Chavez and Song [17] and Dumont et al. [18], which shows that

(45) $$\left\{ \begin{array}{c} \frac{dX_{r}}{dt}=B\left(X_{r}-X_{DFE}\right)+CX_{n},\\ \frac{dX_{n}}{dt}=DX_{n}, \end{array}\right.$$

where $X_{r}$ is the vector of compartments that do not transmit the disease, $X_{n}$ is the vector of all the transmitting compartments, and B, C, and D are matrices to be obtained from model equations. By using this theorem, we get

(46) $$X_{r}=\left(S_{ma},S_{mb},S_{fa},S_{fb},S_{v}\right),$$

(47) $$\begin{array}{ccc} X_{r}-X_{DFE} & = & (S_{ma}-\frac{\lambda (\lambda_{h}N_{h})}{\mu_{h}\left(\mu_{h}+\tau \right)},S_{mb}-\frac{\lambda_{h}N_{h}}{\mu_{h}+\tau },S_{fa}-\frac{\tau \lambda_{h}N_{h}}{\mu_{h}\left(\mu_{h}+\tau \right)},S_{fb}\\ & & -\;\frac{\lambda_{h}N_{h}}{\mu_{h}+\tau },S_{v}-\frac{\lambda_{v}N_{v}}{\mu_{v}+\psi_{v}\alpha_{v}I_{h}}){}^{t}, \end{array}$$

(48) $$\frac{dX_{r}}{dt}=B\left(X_{r}-X_{DFE}\right)+CX_{n}.$$

We have

(49) $$\left[\begin{array}{c} \frac{dS_{ma}}{dt}\\ \frac{dS_{mb}}{dt}\\ \frac{dS_{fa}}{dt}\\ \frac{dS_{fb}}{dt}\\ \frac{dS_{v}}{dt} \end{array}\right]=B\;\left[\begin{array}{c} S_{ma}-\frac{\lambda (\lambda_{h}N_{h})}{\mu_{h}\left(\mu_{h}+\tau \right)}\\ S_{mb}-\frac{\lambda_{h}N_{h}}{\mu_{h}+\tau }\\ S_{fa}-\frac{\tau \lambda_{h}N_{h}}{\mu_{h}\left(\mu_{h}+\tau \right)}\\ S_{fb}-\frac{\lambda_{h}N_{h}}{\mu_{h}+\tau }\\ S_{v}-\frac{\lambda_{v}N_{v}}{\mu_{v}+\psi_{v}\alpha_{v}I_{h}} \end{array}\right]+C\;\left[\begin{array}{c} E_{ma}\\ I_{ma}\\ E_{mb}\\ I_{mb}\\ E_{fa}\\ I_{fa}\\ E_{fb}\\ I_{fb}\\ E_{v}\\ I_{v} \end{array}\right].$$

From equation (49), we obtain

(50) $$\begin{array}{ccc} & & \left[\begin{array}{c} \tau S_{mb}+\theta_{ma}E_{ma}+\xi_{ma}I_{ma}+\gamma_{ma}R_{ma}-\left(\mu_{h}+\psi_{ma}\alpha_{ma}I_{v}\right)S_{ma}\\ \lambda_{h}N_{h}+\theta_{mb}E_{mb}+\xi_{mb}I_{mb}+\gamma_{mb}R_{mb}-\left(\mu_{h}+\tau +\psi_{mb}\alpha_{mb}I_{v}\right)S_{mb}\\ \tau S_{fb}+\theta_{fa}E_{fa}+\xi_{fa}I_{fa}+\gamma_{fa}R_{fa}-\left(\mu_{h}+\psi_{fa}\alpha_{fa}I_{v}\right)S_{fa}\\ \lambda_{h}N_{h}+\theta_{fb}E_{fb}+\xi_{fb}I_{fb}+\gamma_{fb}R_{fb}-\left(\mu_{h}+\tau +\psi_{fb}\alpha_{fb}I_{v}\right)S_{fb}\\ \lambda_{v}N_{v}-\left(\mu_{v}+\psi_{v}\alpha_{v}I_{h}\right)S_{v} \end{array}\right]\\ & & =B\;\left[\begin{array}{c} S_{ma}-\frac{\lambda (\lambda_{h}N_{h})}{\mu_{h}\left(\mu_{h}+\tau \right)}\\ S_{mb}-\frac{\lambda_{h}N_{h}}{\mu_{h}+\tau }\\ S_{fa}-\frac{\tau \lambda_{h}N_{h}}{\mu_{h}\left(\mu_{h}+\tau \right)}\\ S_{fb}-\frac{\lambda_{h}N_{h}}{\mu_{h}+\tau }\\ S_{v}-\frac{\lambda_{v}N_{v}}{\mu_{v}+\psi_{v}\alpha_{v}I_{h}} \end{array}\right]+C\;\left[\begin{array}{c} E_{ma}\\ I_{ma}\\ E_{mb}\\ I_{mb}\\ E_{fa}\\ I_{fa}\\ E_{fb}\\ I_{fb}\\ E_{v}\\ I_{v} \end{array}\right]. \end{array}$$

From equation (50), matrices B and C are found to be given by

(51) $$B=\left[\begin{array}{ccccc} -(\mu_{h}+\psi_{ma}\alpha_{ma}I_{v}) & \tau & 0 & 0 & 0\\ 0 & -(\mu_{h}+\tau +\psi_{mb}\alpha_{mb}I_{v}) & 0 & 0 & 0\\ 0 & 0 & -(\mu_{h}+\psi_{fa}\alpha_{fa}I_{v}) & \tau & 0\\ 0 & 0 & 0 & -(\mu_{h}+\tau +\psi_{fb}\alpha_{fb}I_{v}) & 0\\ 0 & 0 & 0 & 0 & -(\mu_{v}+\psi_{v}\alpha_{v}I_{h}) \end{array}\right],$$

(52) $$C=\left[\begin{array}{cccccccccc} \theta_{ma} & \xi_{ma} & 0 & 0 & 0 & 0 & 0 & 0 & 0 & -\phi_{ma}\alpha_{ma}S_{ma}^{*}\\ 0 & 0 & \theta_{mb} & \xi_{mb} & 0 & 0 & 0 & 0 & 0 & -\phi_{mb}\alpha_{mb}S_{mb}^{*}\\ 0 & 0 & 0 & 0 & \theta_{fa} & \xi_{fa} & 0 & 0 & 0 & -\phi_{fa}\alpha_{fa}S_{fa}^{*}\\ 0 & 0 & 0 & 0 & 0 & 0 & \theta_{fb} & \xi_{fb} & 0 & -\phi_{fb}\alpha_{fb}S_{fb}^{*}\\ 0 & 0 & 0 & 0 & 0 & 0 & 0 & 0 & 0 & 0 \end{array}\right].$$

It can be observed that the matrix B has five eigenvalues, $\lambda_{1}=-\left(\mu_{v}+\psi_{v}\alpha_{v}I_{h}\right),$ $\lambda_{2}=-\left(\mu_{h}+\tau +\psi_{fb}\alpha_{fb}I_{v}\right),$ $\lambda_{3}=-\left(\mu_{h}+\psi_{fa}\alpha_{fa}I_{v}\right),$ $\lambda_{4}=-\left(\mu_{h}+\tau +\psi_{mb}\alpha_{mb}I_{v}\right),$ and $\lambda_{5}=-\left(\mu_{h}+\psi_{ma}\alpha_{ma}I_{v}\right)$ which are real and all negative, which make the system

(53) $$\frac{dX_{r}}{dt}=B\left(X_{r}-X_{DFE}\right)+CX_{n}$$

be globally stable at the disease-free equilibrium point. It remains to show that the matrix D is also stable for the system

(54) $$\frac{dX_{n}}{dt}=DX_{n}.$$

So, we have

(55) $$\left[\begin{array}{c} \psi_{ma}\alpha_{ma}I_{v}S_{ma}+\tau E_{mb}-\left(\mu_{h}+\theta_{ma}+\phi_{ma}\right)E_{ma}\\ \phi_{ma}E_{ma}+\tau I_{mb}-\left(\left(\mu_{h}+\sigma_{ma}\right)+\xi_{ma}+\zeta_{ma}\right)I_{ma}\\ \psi_{mb}\alpha_{mb}I_{v}S_{mb}-\left(\mu_{h}+\theta_{mb}+\tau +\phi_{mb}\right)E_{mb}\\ \phi_{mb}E_{mb}-\left(\left(\mu_{h}+\sigma_{mb}\right)+\xi_{mb}+\tau +\zeta_{mb}\right)I_{mb}\\ \psi_{fa}\alpha_{fa}I_{v}S_{fa}+\tau E_{fb}-\left(\mu_{h}+\theta_{fa}+\phi_{fa}\right)E_{fa}\\ \phi_{fa}E_{fa}+\tau I_{fb}-\left(\left(\mu_{h}+\sigma_{fa}\right)+\xi_{fa}+\zeta_{fa}\right)I_{fa}\\ \psi_{fb}\alpha_{fb}I_{v}S_{fb}-\left(\mu_{h}+\theta_{fb}+\tau +\phi_{fb}\right)E_{fb}\\ \phi_{fb}E_{fb}-\left(\left(\mu_{h}+\sigma_{fb}\right)+\xi_{fb}+\tau +\zeta_{fb}\right)I_{fb}\\ \psi_{v}\alpha_{v}I_{h}S_{v}-\left(\mu_{v}+\phi_{v}\right)E_{v}\\ \phi_{v}E_{v}-\mu_{v}I_{v} \end{array}\right]=D\left[\begin{array}{c} E_{ma}\\ I_{ma}\\ E_{mb}\\ I_{mb}\\ E_{fa}\\ I_{fa}\\ E_{fb}\\ I_{fb}\\ E_{v}\\ I_{v} \end{array}\right].$$

From equation (55), we obtain the matrix D, which is given by

$$\begin{array}{cc} D= & [\begin{array}{ccccc} -(\mu_{h}+\theta_{ma}+\phi_{ma}) & 0 & \tau & 0 & 0\\ \phi_{ma} & -(\left(\mu_{h}+\sigma_{ma}\right)+\xi_{ma}+\zeta_{ma} & 0 & \tau & 0\\ 0 & 0 & -(\mu_{h}+\theta_{mb}+\tau +\phi_{mb}) & 0 & 0\\ 0 & 0 & \phi_{mb} & -(\mu_{h}+\sigma_{mb}+\xi_{mb}+\tau +\zeta_{mb}) & 0\\ 0 & 0 & 0 & 0 & -(\mu_{h}+\theta_{fa}+\phi_{fa})\\ 0 & 0 & 0 & 0 & \phi_{fa}\\ 0 & 0 & 0 & 0 & 0\\ 0 & 0 & 0 & 0 & 0\\ 0 & 0 & 0 & 0 & 0\\ 0 & 0 & 0 & 0 & 0 \end{array}\\ & \begin{array}{ccccc} 0 & 0 & 0 & 0 & \psi_{ma}\alpha_{ma}S_{ma}^{*}\\ 0 & 0 & 0 & 0 & 0\\ 0 & 0 & 0 & 0 & \psi_{mb}\alpha_{mb}S_{mb}^{*}\\ 0 & 0 & 0 & 0 & 0\\ 0 & 0 & 0 & 0 & \psi_{fa}\alpha_{fa}S_{fa}^{*}\\ -(\mu_{h}+\sigma_{fa}+\xi_{fa}+\zeta_{fa}) & 0 & \tau & 0 & 0\\ 0 & -(\mu_{h}+\theta_{fb}+\tau +\phi_{fb}) & 0 & 0 & \psi_{fb}S_{fb}^{*}\\ 0 & \phi_{fb} & -(\mu_{h}+\sigma_{fb}+\xi_{fb}+\tau +\zeta_{fb}) & 0 & 0\\ 0 & 0 & 0 & -(\mu_{v}+\phi_{v}) & 0\\ 0 & 0 & 0 & 0 & -\mu_{v} \end{array}]. \end{array}$$

We observe that the expressions in the main diagonal of the matrix D are all real and negative eigenvalues, which makes the matrix D be globally stable, and so the system is at the disease-free equilibrium point.

## 4 Results and discussion

### 4.1 Numerical results

**Fig. 2:**
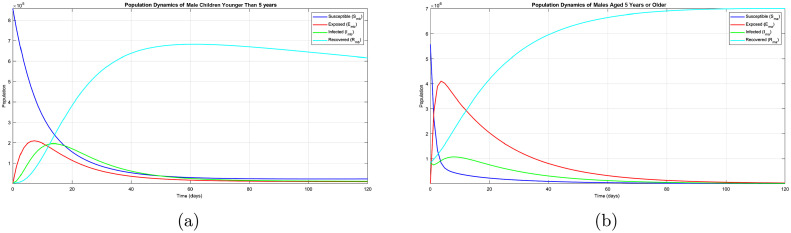
(a) Population of males younger than 5 years. (b) Population of males aged 5 years or older.

**Fig. 3:**
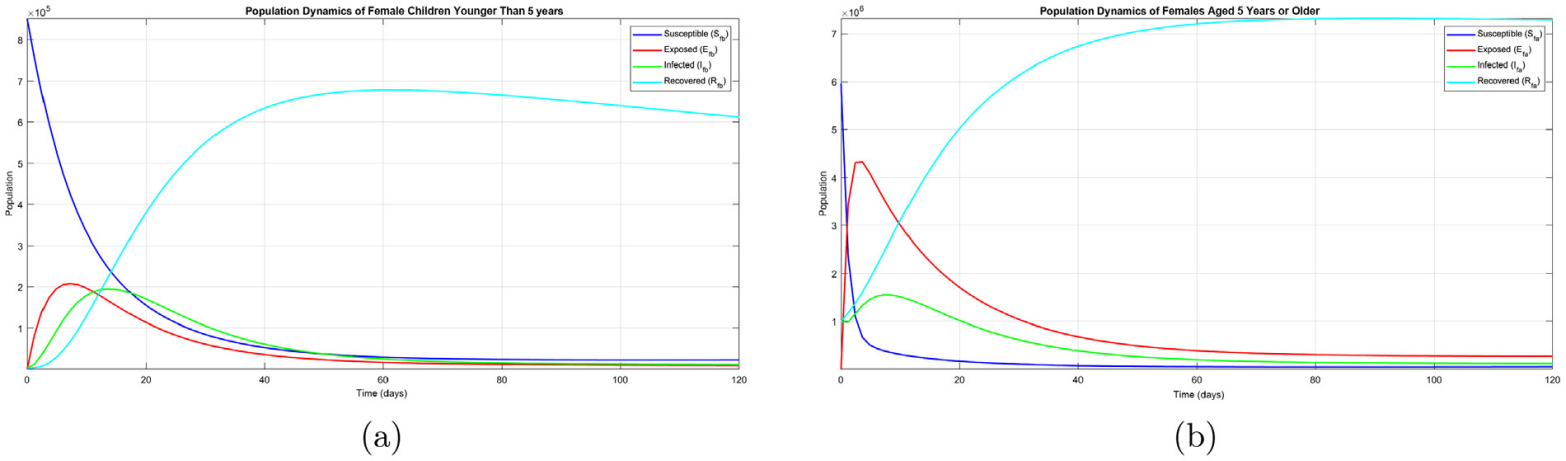
(a) Population of females younger than 5 years. (b) Population of females aged 5 years or older.

**Fig. 4:**
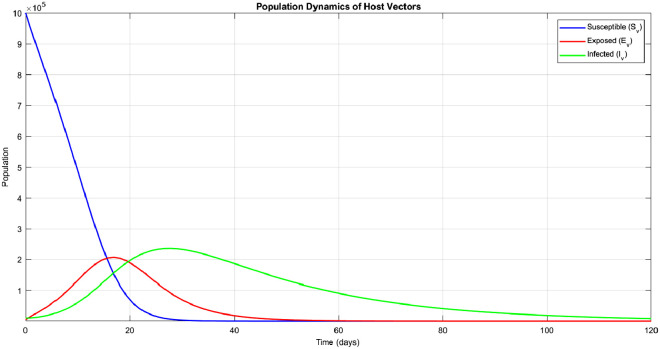
Population of vectors.

The mathematical model that describes the dynamical behavior of malaria was numerically solved. To be able to simulate the model, some parameter literature values were used and others were assumed on the basis of prior knowledge of the disease; they are given in Table 3. The initial solutions used for the simulations are based on real data found in *Rwanda Statistical YearBook 2018* [20], except for data for the infectious classes for 2018, which were obtained from the website of the Rwanda Biomedical Centre. The initial solutions of the model were set to be $X_{0}=\left[S_{mb_{0}},E_{mb_{0}},I_{mb_{0}},R_{mb_{0}},S_{ma_{0}},E_{ma_{0}},I_{ma_{0}},R_{ma_{0}},S_{fb_{0}},E_{fb_{0}},I_{fb_{0}},R_{fb_{0}},S_{fa_{0}},E_{fa_{0}},I_{fa_{0}},R_{fa_{0}},S_{v_{0}},E_{v_{0}},I_{v_{0}}\right]=\left[856228,10,2913,2800,5573098,10,821626,821526,852232,10,2723,2654,5964836,10,993713,993569,1000000,5000,10000\right]$. The numerical results obtained are shown in Figures 2-4.

**Table 3:** Values of the parameters used in the numerical simulation.

Parameter	Value (${\text{day}}^{-1}$)	Source
$\lambda_{h}$	0.000097	Chitnis et al. [19]
$\lambda_{v}$	0.13	Chitnis et al. [19]
$\mu_{h}$	0.00004	Okuneye and Gumel [30]
$\mu_{v}$	0.052	Addawe and Lope [9]
τ	0.000183	Okuneye and Gumel [30]
$\psi_{mb}$	0.35	Assumed
$\psi_{ma}$	0.30	Assumed
$\psi_{fb}$	0.35	Assumed
$\psi_{fa}$	0.32	Assumed
$\psi_{v}$	0.40	Assumed
$\alpha_{mb}$	0.27	Okuneye and Gumel [30]
$\alpha_{ma}$	0.22	Okuneye and Gumel [30]
$\alpha_{fb}$	0.27	Okuneye and Gumel [30]
$\alpha_{fa}$	0.25	Okuneye and Gumel [30]
$\alpha_{v}$	0.30	Assumed
$\theta_{mb}$	0.08	Assumed
$\theta_{ma}$	0.10	Assumed
$\theta_{fb}$	0.08	Assumed
$\theta_{fa}$	0.10	Assumed
$\phi_{mb}$	0.203	Okuneye and Gumel [30]
$\phi_{ma}$	0.07	Okuneye and Gumel [30]
$\phi_{fb}$	0.203	Okuneye and Gumel [30]
$\phi_{fa}$	0.09	Okuneye and Gumel [30]
$\phi_{v}$	0.1	Chitnis et al. [19]
$\xi_{mb}$	0.03	Assumed
$\xi_{ma}$	0.05	Assumed
$\xi_{fb}$	0.03	Assumed
$\xi_{fa}$	0.04	Assumed
$\sigma_{mb}$	0.0000327	Addawe and Lope [9]
$\sigma_{ma}$	0.0000123	Addawe and Lope [9]
$\sigma_{fb}$	0.0000123	Addawe and Lope [9]
$\sigma_{fa}$	0.0000153	Assumed
$\zeta_{mb}$	0.0014	Addawe and Lope [31]
$\zeta_{ma}$	0.0018	Assumed
$\zeta_{fb}$	0.0014	Addawe and Lope [31]
$\zeta_{fa}$	0.0016	Assumed
$\gamma_{mb}$	0.0027	Addawe and Lope [31]
$\gamma_{ma}$	0.0027	Addawe and Lope [9]
$\gamma_{fb}$	0.0027	Addawe and Lope [31]
$\gamma_{fa}$	0.0027	Addawe and Lope [9]

The results in Figure 2(a) show that susceptible male children younger than 5 years are getting infected by contact with the infected host vector and move to the exposed compartment. After some time, the exposed male children show the symptoms of malaria, and a high number of them get treated and and recover. However, a small number of infectious male children younger than 5 years succumbed to malaria.

From Figure 2(b), the number of susceptible males aged 5 years or older decreases as a result of their being infected with malaria and they become asymptomatic, and later, after developing symptoms, they move to the infectious compartment. The number of infected individuals declines as many of them recover from the disease. Therefore, from comparison of Figures 2(a) and 2(b), there is a higher number of infectious male children younger than 5 years than infectious males aged 5 years or older.

The same scenario exists for the female population. From comparison of Figures 3(a) and (b), there is a higher number of exposed and infectious female children younger than 5 years than infectious females aged 5 years or older. From comparison of Figures 2(a) and 3(a), there seems to be no great difference between malaria dynamics in male children younger than 5 years and in female children younger than 5 years. However, a slight difference can be observed between males aged 5 years or older (Figure 2(b)) and females aged 5 years or older (Figure 3(b)).

In Figure 4, we see that the population of susceptible vectors decreases as a result of their biting infectious people, and they move to the exposed class and later become infectious and continue to spread malaria by biting people until they die.

### 4.2 Sensitivity analysis

To compute the degree of the sensitivity of the parameters of the model to the disease’s transmission, we apply the normalized forward sensitivity index method applied by Chitnis et al. [19]. If $\Omega_{i}$ is a parameter in the expression for the basic reproduction number $R_{0}$, then the sensitivity index of $R_{0}$ with respect to $\Omega_{i}$ is obtained as

(56) $$\kappa_{\Omega_{i}}^{R_{0}}=\frac{\partial R_{0}}{\partial \Omega_{i}}\times \frac{\Omega_{i}}{R_{0}}.$$

**Fig. 5:**
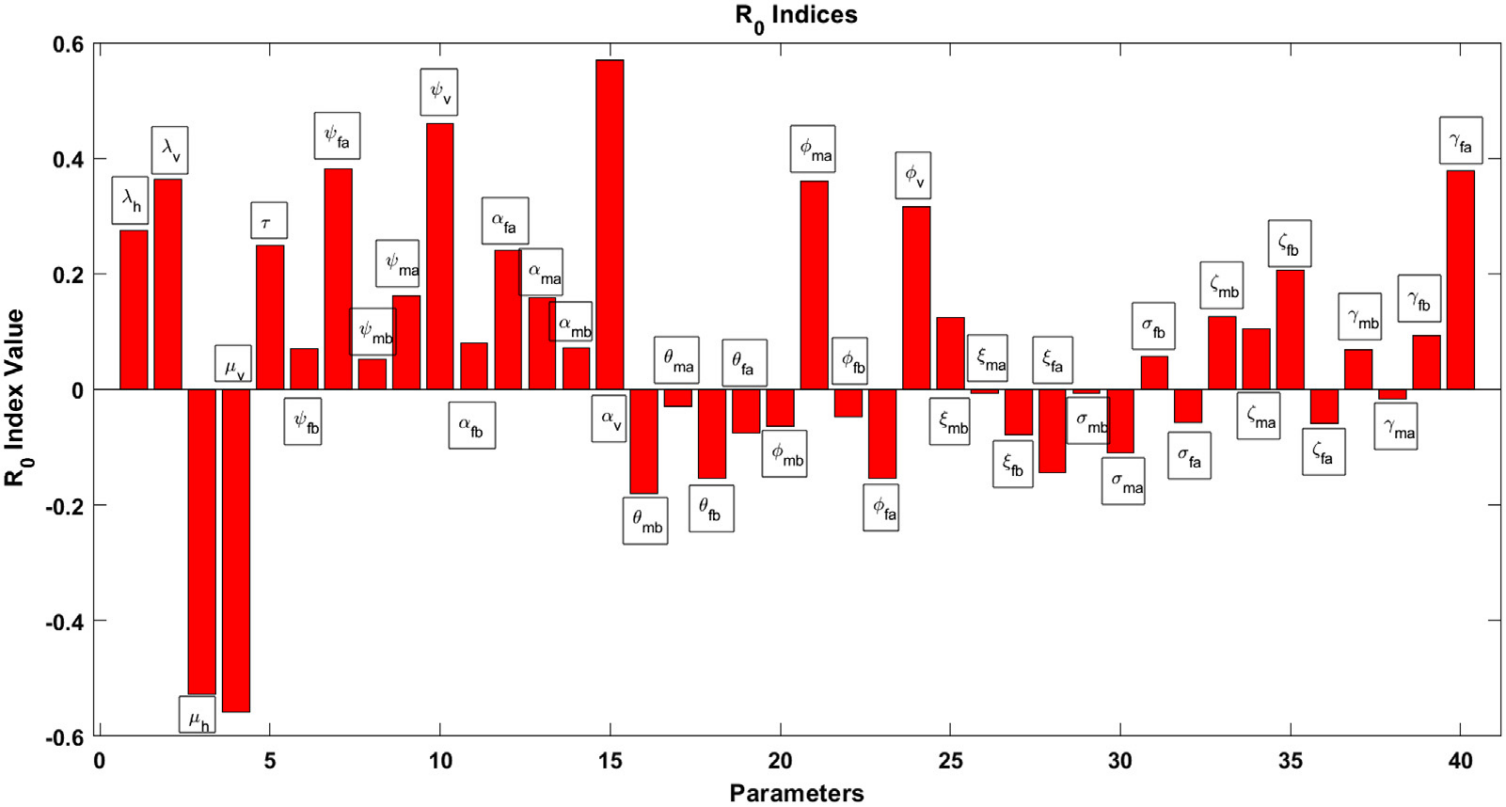
Sensitivity analysis.

By use of this formula, the sensitivity indices are numerically evaluated with use of the parameter values in Table 3, and they are summarized in Table 4; the graphical results are presented in Figure 5.

**Table 4:** Sensitivity analysis of parameters on $R_{0}$.

Parameter	Sensitivity index
$\lambda_{h}$	0.2745
$\lambda_{v}$	0.3639
$\mu_{h}$	−0.5267
$\mu_{v}$	−0.5578
τ	0.2486
$\psi_{fb}$	0.0707
$\psi_{fa}$	0.3820
$\psi_{mb}$	0.0527
$\psi_{ma}$	0.1625
$\psi_{v}$	0.4602
$\alpha_{fb}$	0.0807
$\alpha_{fa}$	0.2402
$\alpha_{mb}$	0.1581
$\alpha_{ma}$	0.0714
$\alpha_{v}$	0.5704
$\theta_{mb}$	−0.1795
$\theta_{ma}$	−0.0287
$\theta_{fb}$	−0.1547
$\theta_{fa}$	−0.0750
$\phi_{mb}$	−0.0633
$\phi_{ma}$	0.3608
$\phi_{fb}$	−0.0482
$\phi_{fa}$	−0.1541
$\phi_{v}$	0.3157
$\xi_{mb}$	0.1247
$\xi_{ma}$	−0.0069
$\xi_{fb}$	−0.0785
$\xi_{fa}$	−0.1445
$\sigma_{mb}$	−0.0063
$\sigma_{ma}$	−0.1094
$\sigma_{fb}$	0.0570
$\sigma_{fa}$	−0.0581
$\zeta_{mb}$	0.1263
$\zeta_{ma}$	0.1049
$\zeta_{fb}$	0.2067
$\zeta_{fa}$	−0.0586
$\gamma_{mb}$	0.0684
$\gamma_{ma}$	−0.0170
$\gamma_{fb}$	0.0932
$\gamma_{fa}$	0.3779

Replacing these indices in the analytical results of overall $R_{0}$ and partial $R_{0}$ in equation (44), we obtained the following results. The overall basic reproduction number $R_{0}$ is 1.9247. The partial basic reproduction numbers for males younger than 5 years, males aged 5 years or older, females younger than 5 years, and females aged 5 years or older are, respectively, $R_{0mb}=0.5082$, $R_{0ma}=0.0232$, $R_{0fb}=0.3649$, and $R_{0fa}=2.8081.$

The sensitivity index for each parameter is obtained by the use of equation (56) and the parameter values given in Table 3. If the sensitivity index has a positive sign, this implies that an increase or a decrease of the parameter value when the other parameters are kept constant will increase or decrease the value of the basic reproduction number $R_{0}$. On the other hand, a negative sign of the sensitivity index indicates that an increase or a decrease of the parameter value will decrease or increase the value of the basic reproduction number. Hence, $R_{0}$ is an increasing or a decreasing function of a positive or negative parameter, respectively. The sensitivity indices show how sensitive $R_{0}$ is to the parameter. For instance, if the infection rate of females aged 5 years or older ($\psi_{fa}$) is increased by 10%, the basic reproduction number will increase by 3.82%, which is an indication of disease transmission. Likewise, if the death rate of vectors ($\mu_{v}$) is increased by 10%, the basic reproduction number will decrease by 5.58%, which is a noticeable decrease in disease transmission.

The most positive sensitive parameters in the model are the force of infection for vectors ($\alpha_{v}$), the infection rate for vectors ($\psi_{v}$), the infection rate for females aged 5 years or older ($\psi_{fa}$), the per capita birth rate of vectors ($\lambda_{v}$), and the rate of recovered females who may lose immunity and move back to the susceptible class ($\gamma_{fa}$). The most negative sensitive parameters in the model are the per capita death rate of vectors ($\mu_{v}$) and the per capita death rate of humans ($\mu_{h}$). However, as it is unethical and not practical to increase human natural mortality; for control of malaria, the per capita death rate of vectors must be considered, meaning that to decrease $R_{0}$, the per capita death rate of vectors must be increased.

### 4.3 Discussion

By comparing results obtained in this study with other results, we find agreement with the most positive sensitive parameters of Kalula et al. [10], according to whom the most positive sensitive parameter was the biting rate, which is similar to the infection rate and the force of infection together in our case as the infection rate is influenced by the biting rate and the force of infection. Similar results were obtained by Chitnis et al. [19]. In addition, we found that the infection rate for females aged 5 years or older is among the positive sensitive parameters. Likewise, the most negative sensitive parameters are the same in our study as in the study of Kalula et al. [10], being the per capita death rate of vectors ($\mu_{v}$) and the per capita death rate of humans ($\mu_{h}$). We obtained the disease-free equilibrium point in accordance with [9], [10], [14].

On the basis of the findings of this study, it is revealed that there is no difference between the infection rates of males and females younger than 5 years because infected mosquitoes do not choose which sex to bite and infect. This is consistent with the study of Mbanugo and Ejims [21], who found that sex has no effect on malaria incidence among children younger than 5 years. Thus, it fits with the findings of Olasunkanmi et al. [22], who reported that malaria can kill people of all ages and both sexes.

Nevertheless, Mbishi et al. [23] concluded that children younger than 5 years regardless of sex have high morbidity and high malaria risk because of different factors in sub-Saharan African countries, such as maternal education and sulfadoxine-pyrimethamine uptake during pregnancy. In addition, the increase in the infection rate of children younger than 5 years is positively associated with an increase in age as highlighted by several researchers [24], [25], [26], and malaria is highly linked to different factors, including playing outdoors, children’s indoor activities that increase their exposure to biting by mosquitoes, and other factors, such as anemia, socioeconomic factors such as residence type, toilet facilities, wealth index, the use of electricity, mothers’ education, and children’s age, and geographical factors such as the region and the altitude of the region of residence. However, those researchers did not analyze and compare age groups, while this study compared malaria prevalence between children younger than 5 years and people aged 5 years or older taking into account sex stratification and found that the rate rises with age, which means that they are all at risk of malaria but those aged 5 years or older are at higher risk than children younger than 5 years.

The incidence of malaria was found to be higher among males and females aged 5 years or older than among children younger than 5 years, and this finding is in agreement with the study of Coulibaly et al. [27], who recommended that there should be some measures to control malaria among older children. The main reasons behind children aged 5 years or older being infected with malaria could be their attending schools, their playing around stagnant pools of water and bushes, which are breeding sites of *Plasmodium falciparum*, their tendency to be independent from the shielding and special care of their parents, and their behavior in not complying with parents’ preventive measures against malaria.

On the other hand, this study has shed light on how malaria is impacted by sex. Females are more likely affected by malaria and are more susceptible to it, and this finding is consistent with a study conducted in Uganda from January 2020 through April 2021 at 12 public health facilities by Okiring et al. [6], who pointed out that the incidence of malaria for females is almost twice that for males. This is also in line with the study of Quaresima et al. [28], who revealed that women in Ghana are more vulnerable than men (62% versus 42%). Ghanaian women’s responsibilities expose them to mosquito bites in dangerous hours such as after sunset while doing business and fetching water before sunrise, while Ugandan women always care for their households, which exposes them to mosquito bites, and they often go to healthcare centers not for the care of children, for febrile reasons but also for antenatal care, which resulted in researchers having more data about women than men.

Our study has ended any misunderstanding about who is at high risk of malaria regarding sex. The main reason behind this is attributed to the existing social-based Rwandan culture, which dictates that women in Rwanda are the ones who collect water and firewood in the forest, wash the clothes of the family, cook almost every meal for the family, clean the household compound, are supposed to find cleaning materials outdoors, take care of cattle at home, and participate with their husbands in agricultural activities but to a greater extent than the men, including irrigation, and take children to the health center, where they meet infected people and where infected *Plasmodium* can bite them. Those different socioeconomic and cultural responsibilities make women more exposed to the mosquito’s bite.

In contrast to these findings, Tanzanian men are more affected by malaria than Tanzanian women because of their spending many hours outdoors in the evening drinking alcohol, watching soccer and movies on television, and sharing ideas, which expose them to the high biting rate of mosquitoes [29]. Tanzanian men’s behavior is in line with Rwandan men’s behavior, but still Rwandan women are more exposed to the high biting rate of mosquitoes due to women’s hardships and responsibilities. Thus, it is reasonable and understandable that when the biting rate increases, there is also a high probability of an increased infection rate, which leads to increased malaria incidence. This study’s objective was not to examine the factors behind the difference in infection rate regarding sex and age and the incidence of malaria among Rwandans, but we cannot ignore socioeconomic and cultural aspects of Rwandans that are behind the decision-making of families regarding normal life, economic development, and behavior among Rwandan families from the past until now.

## 5 Conclusion and recommendations

This study’s objective was to develop and analyze the malaria transmission model taking into account the age-sex structure of the population and recommend potential interventions, mechanisms, and methods to fight against the prevalence of malaria in Rwanda. It is found that the prevalence of malaria among children younger than 5 years is less than that among people aged 5 years or older and that the incidence of malaria increases in accordance with the increase in age. There is no difference in malaria incidence between the sexes among children younger than 5 years. From the findings of this study, among people aged 5 years or older, females are more affected by malaria than males. Practically, these findings indicate how far females should wake up than before this study, be careful and seek more knowledge regarding malaria, and try their best to protect themselves against malaria. Additionally, females are the ones who in Rwanda generally take care of the children. They must think about how to comply with governmental preventive measures against malaria to protect the whole family, for example, by educating their children aged 5 years or older where to play their games to ensure that the place is not close to any breeding site for mosquitoes, by ensuring that children younger than 5 years sleep under a bed net, and by ensuring that the household is well cleaned and that no breeding site for mosquitoes is close to the house. Finally, because of women’s responsibilities for their households, the government needs to ensure that outdoor spraying is effective to impede the speed with which mosquitoes are infected and infect people. The model can be extended to include climatic factors such as temperature, rainfall, and humidity as they have a significant effect on malaria transmission.

## Funding source

This work was funded by a grant from the 10.13039/501100007114African Institute for Mathematical Sciences (IDRC Grant No: 108246-001), with financial support from the Government of Canada, provided through Global Affairs Canada and the International Development Research Centre.

## Ethical approval

The data used in this work are open source and accessible from the websites of the Rwanda Biomedical Centre (https://rbc.gov.rw/welcome-to-rbc) and the National Institute of Statistics of Rwanda (https://www.statistics.gov.rw/).

## Declaration of competing interest

The authors declare that they have no known competing financial interests or personal relationships that could have appeared to influence the work reported in this article.
